# Association of *Anopheles sinensis* average abundance and climate factors: Use of mosquito surveillance data in Goyang, Korea

**DOI:** 10.1371/journal.pone.0244479

**Published:** 2020-12-28

**Authors:** Jin Young Jang, Byung Chul Chun

**Affiliations:** 1 Department of Public Health, Korea University Graduate School, Seoul, Korea; 2 Department of Preventive Medicine, Korea University College of Medicine, Seoul, Korea; Columbia University, UNITED STATES

## Abstract

Malaria is a vector-borne disease transmitted by *Anopheline* mosquitoes. In Korea, *Plasmodium vivax* malaria is an endemic disease and the main vector is *Anopheles sinensis*. *Plasmodium vivax* malaria is common in the northwestern part of South Korea, including in the city of Goyang in regions near the demilitarized zone. This study aimed to identify the best time-series model for predicting mosquito average abundance in Goyang, Korea. Mosquito data were obtained from the Mosquito Surveillance Program of the Goyang Ilsanseogu Public Health Center for the period 2008–2012. Black light traps were set up periodically in a park, a senior community center, and a village community center, public health center, drainage pumping station, cactus research center, restaurant near forest, in which many activities occur at night. In total, 9,512 female mosquitoes were collected at 12 permanent trapping sites during the mosquito season in the study period. Weekly *An*. *sinensis* average abundance was positively correlated with minimum grass temperature (*r* = 0.694, p < 0.001), precipitation (*r* = 0.326, p = 0.001). The results showed that seasonal autoregressive integrated moving average (SARIMA) (1,0,0)(0,0,1)_21_ with minimum grass temperature variable at time lag0 weeks and the precipitation variable at time lag1 weeks provided that best model of mosquito average abundance. The multivariate model accounted for about 54.1% of the mosquito average abundance variation. Time-series analysis of mosquito average abundance and climate factors provided basic information for predicting the occurrence of malaria mosquitoes.

## Introduction

Malaria is a vector-borne disease transmitted by *Anopheline* mosquitoes [1] and caused by parasites of the genus *Plasmodium* (*P*. *vivax*, *P*. *falciparum*, *P*. *ovale*, *P*. *malariae*). The World Health Organization estimated that 219 million clinical episodes (95% confidence interval [CI]; 203–262 million) occurred in 2017 [2]. Korea was considered to be a malaria-free region after two cases in 1984, but *Plasmodium vivax* malaria has re-emerged in Paju (near the Demilitarized Zone), Gyeonggi Province, following one case in a Korean Army soldier in 1993 [3]. After 1993, it peaked at 4,142 cases in 2000. The trend decreased between 2008 (1,052 cases) and 2012 (542 cases). Until recently, endemic malaria cases remained almost constant (mean; 586.43, sd; 90.177) [4, 5]. The major malaria transmitting mosquitoes in Korea, genus *Anopheles*, are distributed in all parts of Asia, such as China and Japan. There are known 8 species of genus *Anopheles* and the main dominant species is *Anopheles sinensis* (Wiedemann) [3, 6].

*Plasmodium vivax* malaria commonly occurs in the northwestern part of the South Korea, including in the city of Goyang in regions near the demilitarized zone (DMZ) [7]. The Korea Centers for Disease Control and Prevention selects an area with at least one patient in the previous year as a malaria-risk area, and this is the case of Goyang [3]. In Korea, there has been no rapid increase in the incidence of malaria in recent years, but there is potential for epidemic because of the uncontrolled geographic location near DMZ.

Vector-borne diseases are very sensitive to climate change. Mosquitoes are ectothermic insects that pass through three aquatic juvenile stages (eggs, larvae, and pupae). Additionally, malarial parasites undergo a development phase in the mosquito. Therefore, mosquito average abundance and climate factors are important determinants of malaria transmission [8, 9].

Previous studies have indicated that temperature, relative humidity, and precipitation are correlated with *An*. *sinensis* average abundance. In Korea, the monthly average temperature and monthly amount of precipitation were significantly correlated with average abundance of mosquitoes on Jeju Island [10]. In China, mosquito average abundance was significantly correlated with the monthly average temperature (*r* = 0.359, p < 0.05) and monthly average relative humidity (*r* = 0.850, p < 0.05) at the Three Gorges Reservoir; with the monthly average temperature (*r* = 0.958, p < 0.001), monthly average relative humidity (*r* = 0.746, p = 0.005), and monthly average precipitation (*r* = 0.725, p = 0.008) at Ningbo city; and the daily average relative humidity (*r* = 0.859, p = 0.029) at Yongcheng city [11–13].

Through these previous studies, we assumed that there was a strong association between *An*. *sinensis* average abundance and climate factors; therefore, we applied a time-series seasonal autoregressive integrated moving average (SARIMA) model for *An*. *sinensis* average abundance prediction in Goyang, Korea. However, the meteorological factors that affect mosquito average abundance may vary by latitude or region, and little research has been performed in Korea, where malaria is prevalent only in the northern region close to the DMZ.

In this study, we aimed to identify the association of using climate factors as independent variables for *Plasmodium vivax* malaria mosquito average abundance in Goyang, Korea, through a 5-year cross-sectional study to find the best model for predicting mosquito average abundance to inform vector control for malaria.

## Materials and methods

### Study area

Goyang (37°34´–37°44´N, 126°40´–126°59´E) is a city in Gyeonggi Province in the northwest of South Korea. It is adjacent to Seoul and downstream of the Han River, and comprises an area of 268.05 km^2^, with three districts (Ilsandong-gu, Ilsanseo-gu, Deogyang-gu). It has a population of over 1 million individuals [14]. Korea has a temperate climate, is influenced by the East Asian monsoon, and has four distinct seasons (spring, summer, autumn, and winter). The average annual rainfall is around 1,300 mm, and is especially heavy in July and August because of a short rainy season and the occurrence of typhoons [15].

### Data source

Mosquito data were obtained from the Mosquito Surveillance Program of the Goyang Ilsanseogu Public Health Center for the period 2008–2012. Monitoring data was collected five times a week from 2008 to 2012 and three times a week in 2013 (excluding public holidays). Due to differences in collection frequency, mosquito average abundance was standardized to the mean number of females per trap per night per week.

**Fig 1:**
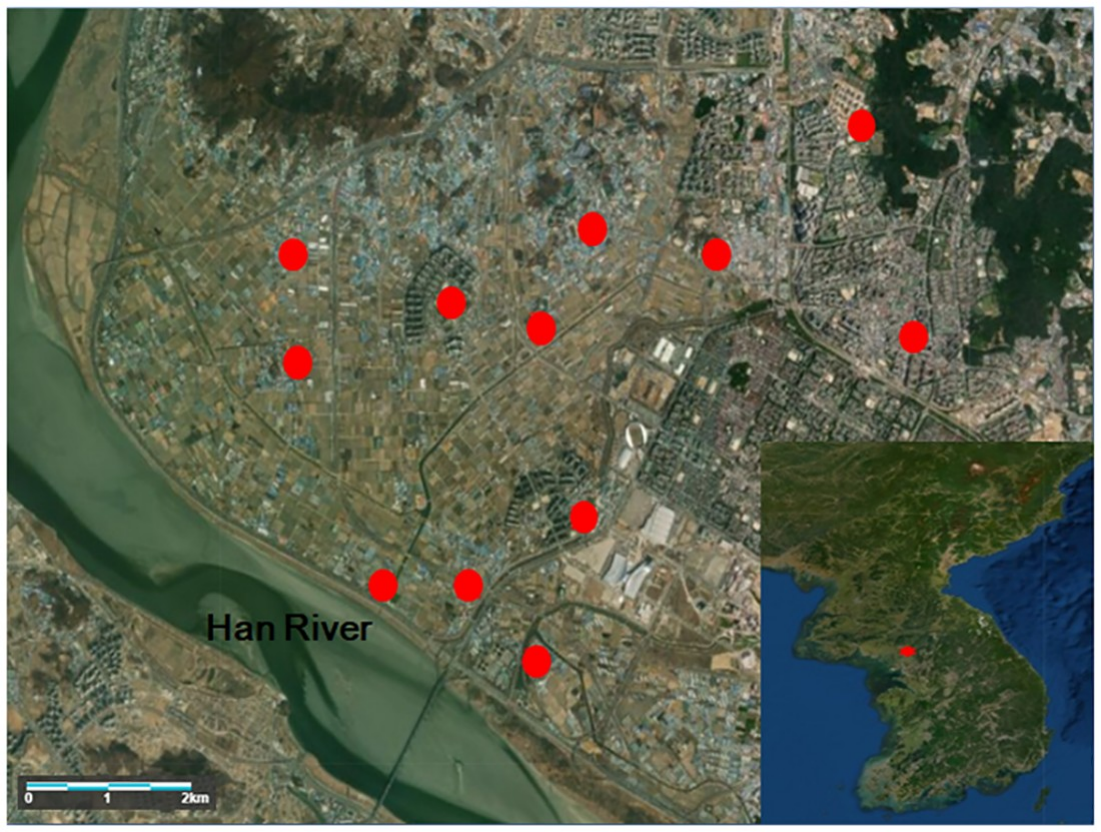
Locations of black light traps at 12 sites in Goyang, Korea, 2008–2012 (copyright map: Https://landlook.usgs.gov/viewer.html).

Black light traps were set up periodically in a park, a senior community center, and a village community center, in which many activities occur at night. Black light trap (model ‘Black Hole’ by BioTrap, http://www.bio-trap.com) is a device that attracts mosquitoes through light generated from UV lamps and carbon dioxide generated by the catalysis of titanium dioxide (TiO2), and the built-in suction fan captures the mosquitoes. Traps were installed at 12 sites (Fig 1) and were operated from 7 p.m. to 6 a.m. throughout the mosquito season. The mosquito season lasts approximately 20 weeks from June to October. We used the data with a continuous time series with a cycle of 21 weeks, there are up to 31–32 weeks intervals between last and first collection between years.

Mosquito classification was performed after freezing at -20°C for 1 day. Mosquitos were identified by morphology, and at a species level according to the manual of Korea Centers for Disease Control and Prevention [16].

Climate data from the Korea Meteorological Administration were used in our study [17]. Independent variables included mean temperature (°C), maximum temperature (°C), minimum temperature (°C), minimum grass temperature (°C), surface temperature (°C), daily temperature range (°C), duration of sunshine (h), relative humidity (%), minimum relative humidity (%), precipitation (mm), mean wind speed (m/s), maximum wind speed (m/s), and maximum instantaneous wind speed (m/s).

It is an open national mosquito surveillance system. No specific permissions were required for this mosquito surveillance activities in this Goyang area by the public health center. The first author participated directly in the data collection and analysis as a public health officer working for this area. We confirm that this field study did not involve endangered or protected species.

### Statistical analysis

Mosquito average abundance and climate data were summarized as the means and standard deviations obtained over 5 years based on 23–43 weeks per year. Time series plots were used to present the distribution of mosquito average abundance and climatic factors. Spearman correlation analysis was performed to identify linear relationships between the various variables.

Before carrying out the time series analysis, the distribution of the data was graphically explored in a histogram. Then, the degree of variance, trend, and seasonality were confirmed using a time-series plot. Season length in the time series was 21 cycles for a total of 105 weeks. Cross-correlation analysis was performed to determine the degree and direction of the relationship between the two variables, and the maximum time lag was 7 previous weeks considering the survival period of mosquitoes. Using the cross-correlation method, we identified significant time lags between mosquito average abundance and climate factors. Significant time lags of climate variables were confirmed through the cross-correlation function plot and fitted as independent variables to the predictive model.

In general, there are three steps in using a seasonal autoregressive integrated moving average (SARIMA) model: Identification, estimation and diagnostic checking [18]. The first step is to look at the patterns of autocorrelation function (ACF) plot and partial autocorrelation function (PACF) plot and to identify preliminary values of order. The parameter estimates are obtained from the maximum likelihood estimation method. The diagnostic checking involves performing the Ljung-Box test to identify the independence of the residuals. In our study, the model with the smallest value of Bayesian information criterion (BIC) is judged as the most appropriate one. The structure of the SARIMA model followed the standard form, (p,d,q)(P,D,Q)s, where p is the order of autoregression; d, the degree of differencing; q, the order of the moving average; P, the seasonal autoregression; D, the degree of seasonal differences; Q, the seasonal moving average; and s, the seasonal period. Multivariate time series analysis was carried out using climate variables as valid independent variables. The model was fitted with all possible combinations of the climate variables with significant lag time obtained through cross-correlation analysis, through a step-wise procedure, and a model with both low Bayesian information criterion (BIC) and high R-square values was selected as the final model. Statistical analyses were performed using SPSS version 21.0 (SPSS Inc, Chicago, IL) and significance was determined at a p-value < 0.05.

## Results

In total, 9,512 mosquitoes were collected from twelve permanent trapping sites during the study period in the mosquito season in 2008–2012 (Table 1). The average number of female mosquitoes captured per trap per night was 7.55 (SD; 10.38), ranging from 0 to 70.7. Weekly mean temperature, minimum grass temperature, and precipitation were 21°C (SD; 4.63), 14.9°C (SD; 6.49), and 8.7 mm (SD; 13.57), respectively (Table 2).

**Table 1:** Total number of *Anopheles sinensis* collected by black light traps at 12 sites in Goyang, Korea, during the period from 23 to 43 weeks in 2008–2012 (n = 9,512).

Year	Mosquitoes, n	%
2008	3,524	37.0
2009	2,180	22.9
2010	1,232	13.0
2011	924	9.7
2012	1,652	17.4

**Table 2:** Distribution of weekly data on *Anopheles sinensis* average abundance and climate factors in Goyang, Korea, during the period from 23 to 43 weeks in 2008–2012.

Weekly data	Mean	SD	Min	P25	P50	P75	Max
**Mosquito average abundance**	7.5	10.38	0	0.5	3.8	12.1	70.7
**Climate factors**							
Mean temperature (°C)	21	4.63	7	18.1	22.7	24.5	28.7
Maximum temperature (°C)	26.9	3.8	15	24.4	27.8	29.5	34.3
Minimum temperature (°C)	16.6	5.59	0.8	13.1	17.9	21	24.1
Minimum grass temperature (°C)	14.9	6.49	-1.6	10.6	16.9	20.1	23.5
Surface temperature (°C)	24.4	5.22	8.8	20.8	25.6	28.1	33.4
Daily temperature range (°C)	10.3	2.73	3.9	8.3	10.3	12.1	16.2
Duration of sunshine (h)	5.7	2.43	0.2	4	5.5	7.7	10.7
Relative humidity (%)	80.3	7.04	55.5	76.4	80.4	85	96.6
Minimum relative humidity (%)	52.1	13.18	22.4	42.8	50.6	61	88.7
Precipitation (mm)	8.7	13.57	0	0.5	2.4	10.9	85.3
Mean wind speed (m/s)	1.4	0.32	0.9	1.2	1.4	1.6	2.3
Maximum wind speed (m/s)	3.6	0.47	2.5	3.3	3.6	3.9	4.8
Maximum instantaneous wind speed (m/s)	6.5	0.94	4.5	5.8	6.5	7.1	9.3

SD: standard deviation; Min: minimum; P: percentile; Max: maximum.

**Fig 2:**
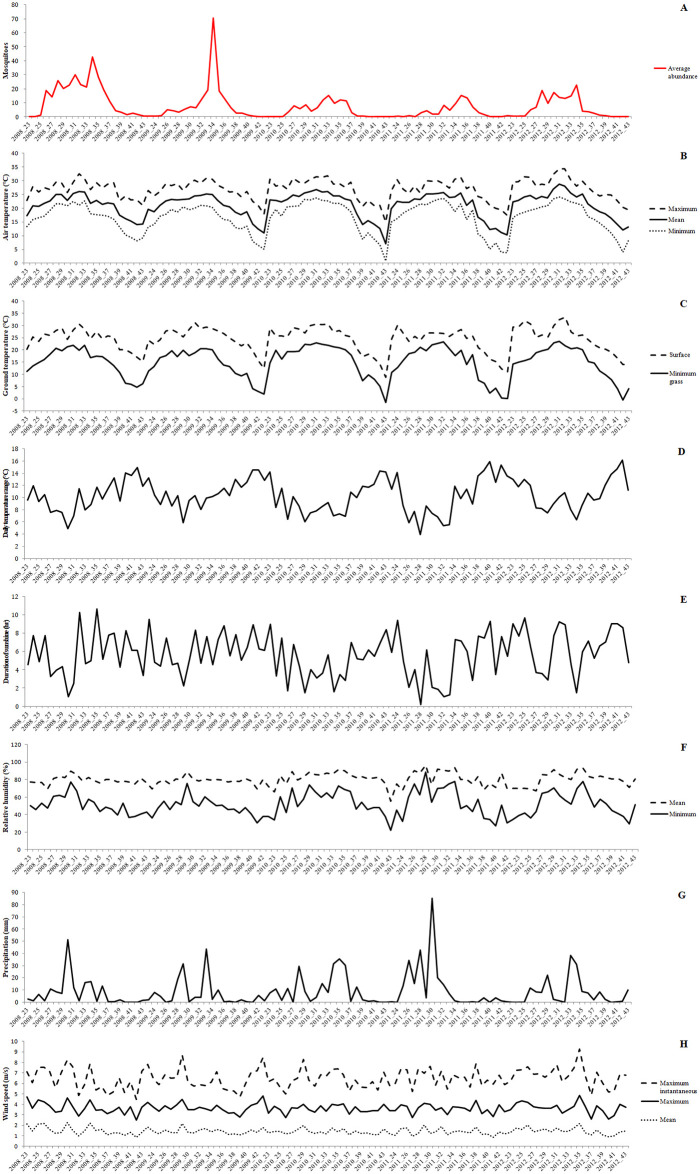
Time series plots of weekly *Anopheles sinensis* average abundance from 2008 to 2012 in Goyang, Korea. A: Mosquito average abundance, B: Air temperature (°C), C: Ground temperature (°C), D: Daily temperature range (°C), E: Duration of sunshine (h), F: Relative humidity(%), G: Precipitation (mm), H: wind speed (m/s).

The mosquito average abundance peaked at 42.83 in week 34 of 2008; 70.67 in week 34 of 2009; 15.42 in week 33 of 2010; 15.33 in week 35 of 2011; and 22.83 in week 34 of 2012 (Fig 2).

Relationships were identified between climate factors and mosquito average abundance. The results of the Spearman correlation analysis to select the primary parameters to fit the model among the climate variables are as follows. *Anopheles sinensis* average abundance was positively correlated with minimum grass temperature (*r* = 0.694, p < 0.001), minimum temperature (*r* = 0.682, p < 0.001), mean temperature (*r* = 0.681, p < 0.001), surface temperature (*r* = 0.593, p < 0.001), maximum temperature (*r* = 0.591, p < 0.001). minimum relative humidity (*r* = 0.509, p < 0.001), relative humidity (*r* = 0.364, p < 0.001), precipitation (*r* = 0.326, p = 0.001), and mean wind speed (*r* = 0.255, p = 0.009). Mosquito average abundance was negatively correlated with the daily temperature range (*r* = -0.535, p < 0.001) and the duration of sunshine (*r* = -0.239, p = 0.014). There was no significant relationship between mosquito average abundance and maximum wind speed (*r* = 0.042, p = 0.67) or maximum instantaneous wind speed (*r* = 0.111, p = 0.26) (Table 3).

**Table 3:** Spearman’s correlations between *Anopheles sinensis* average abundance and climate factors in Goyang, Korea, from 23 to 43 weeks in 2008–2012.

Variable	Mosquitoaverage abundance	Tmean	Tmax	Tmin	Tgrass	Tsurface	DTR	DS	RH	RHmin	Precipita-tion	WSmean	WSmax
**Tmean**	0.681**	–											
**Tmax**	0.591**	0.916**	–										
**Tmin**	0.682**	0.971**	0.819**	–									
**Tgrass**	0.694**	0.946**	0.781**	0.988**	–								
**Tsurface**	0.593**	0.891**	0.952**	0.805**	0.766**	–							
**DTR**	-0.535**	-0.684**	-0.391**	-0.813**	-0.835**	-0.426**	–						
**DS**	-0.239*	-0.293*	0.027	-0.450**	-0.487**	-0.006	0.801**	–					
**RH**	0.364**	0.490**	0.210*	0.627**	0.656**	0.179	-0.780**	-0.742**	–				
**RHmin**	0.509**	0.665**	0.362**	0.795**	0.818**	0.383**	-0.967**	-0.804**	0.878**	–			
**Precipita-tion**	0.326*	0.429**	0.188	0.531**	0.566**	0.207*	-0.735**	-0.688**	0.649**	0.759**	–		
**WSmean**	0.255*	0.245*	0.155	0.274*	0.284*	0.210*	-0.324*	-0.134	-0.040	0.260*	0.351**	–	
**WSmax**	0.042	0.068	-0.002	0.096	0.103	0.047	-0.179	-0.108	-0.109	0.128	0.303*	0.829**	–
**WSinstan-taneous**	0.111	0.173	0.044	0.222*	0.218*	0.097	-0.289*	-0.232*	0.068	0.264*	0.381**	0.757**	0.889**

*Bold values indicate statistically significant differences (ie, *: p < 0.05; **: p < 0.001).

Tmean: mean temperature; Tmax: maximum temperature; Tmin: minimum temperature; Tgrass: minimum grass temperature; Tsurface: surface temperature; DTR: daily temperature range; DS: duration of sunshine; RH: relative humidity; RHmin: minimum relative humidity; WSmean: mean wind speed; WSmax: maximum wind speed; WSinstantaneous: maximum instantaneous wind speed.

**Fig 3:**
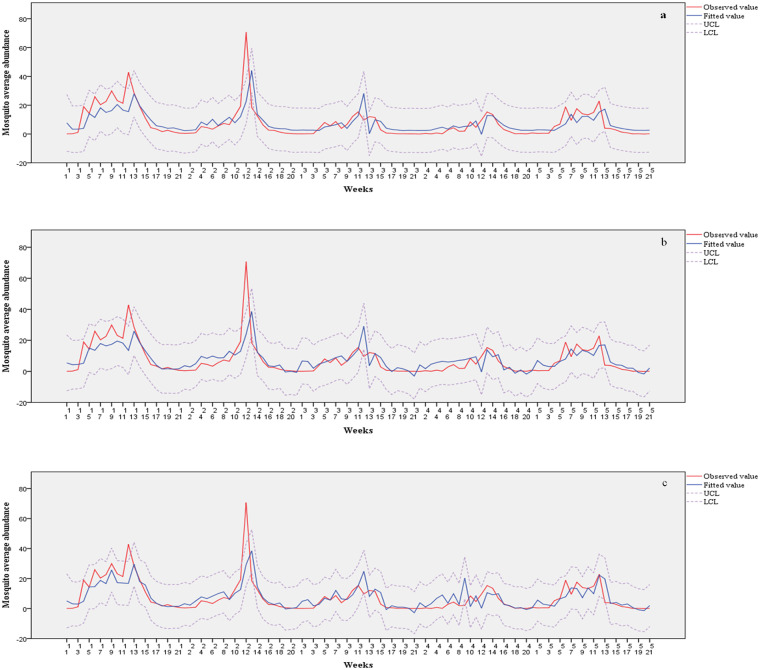
Fitted values of model and observed values for *Anopheles sinensis* average abundance in Goyang, Korea, 2008–2012. a: Model 0, b: Model 1 c: Model 2.

Cross-correlation analysis was performed to identify a significant time lag for all climate factors (S1 Dataset). Then, those variables were sequentially fit on SARIMA (1,0,0)(0,0,1)_21_. *Anopheles sinensis* average abundance and climate factors were fit to three models (Table 4). Model 0, the null model, did not fit the climate variables and was statistically significant at autoregression 1 and seasonal moving average 1. In the model, the residual variance was 0.945 and the R-square value was 0.44. Model 1, the best fitting univariate model, fit the minimum grass temperature variable with a lag of 0 weeks as an independent variable. The residual variance was 0.977 and the R-square value was 0.479. Model 2, the best fitting multivariate model, fit the minimum grass temperature variable with a lag of 0 weeks and the precipitation variable with a lag of 1 week as an independent variable. The residual variance was 0.96 and the R-square value was 0.541 (Fig 3).

**Table 4:** Multivariate seasonal ARIMA models for *Anopheles sinensis* average abundance and climate factors in Goyang, Korea, 2008–2012.

	Model 0			Model 1			Model 2		
	Coefficient	SE	p value	Coefficient	SE	p value	Coefficient	SE	p value
**Autoregression 1**	0.575	0.082	<0.001	0.491	0.088	<0.001	0.583	0.081	<0.001
**Seasonal moving average 1**	-0.335	0.106	0.002	-0.341	0.106	0.002	-0.301	0.109	0.007
**Minimum grass temperature lag 0**	–	–	–	0.620	0.211	0.004	0.476	0.210	0.026
**Precipitation lag 1**	–	–	–	–	–	–	0.178	0.048	<0.001
**Ljung-Box Q**	8.130		0.945	6.796		0.977	7.584		0.960
**R-square**	0.440			0.479			0.541		
**BIC**	4.253			4.235			4.161		

SE: standard error; BIC: Bayesian information criterion.

## Discussion

In this study, we identified relationships between *An*. *sinensis* average abundance and climate factors during mosquito seasons (23–43 weeks, 2008–2012) in Goyang, Korea. In a multivariate model with the minimum grass temperature variable at a lag of 0 weeks, and the precipitation variable at a lag of 1 week, approximately 54.1% of *An*. *sinensis* average abundance variation was explained.

The minimum grass temperature is the lowest temperature reached overnight by a thermometer touching short turf, which is measured at 25–50 mm above the ground and is known as ground frost. Unlike minimum grass temperature, air temperature is measured at 1.25–2 m above the ground [19]. A limited number of studies have investigated the relationship between vector-borne diseases and grass temperature. West Nile virus vector number was positively correlated with grass temperature range (*r* = 0.418, p = 0.003) by Pearson correlation analysis in Cook County, USA. Furthermore, maximum grass temperature was found to be a potential predictor in regression models [20]. In our study, there was a positive correlation with minimum grass temperature (*r* = 0.694, p < 0.001) and a predictor variable in SARIMA model.

Mosquitoes are ectothermic insects, whose body temperature relies on external heat sources, such as ambient temperature. Temperature plays an important role in the survival rate of adult mosquitoes and the mortality rate of the juvenile stage [9]. Furthermore, the main habitat of mosquitoes is forests, wetlands, tall grasses, weeds, and ground. Also, the immature stages of mosquitoes need water habitats. The small pools of water created by the rains provides habitat for eggs, lavas and pupas to develop. Excess precipitation may reduce mosquito abundance through flushing effect. However, the number and size of breeding habitats generally associated with the amount of precipitation [21]. Therefore, given these relationships between temperature, precipitation, and mosquito biology, it is not surprising that grass temperature and precipitation were important predictors of *An*. *sinensis* average abundance.

The present study was not without limitations. We collected mosquito data at 12 sites in Goyang, Korea. Our study collected mosquitoes in urban areas where there are many night activities of people, not near cowsheds or pigstys. *An*. *sinensis* was attracted to cows, pigs and dogs more than humans [22]. Also, mosquito collection may be affected near tump or streams. So, to reduce the spatial variability, we standardized to mosquito average abundance, not the number of mosquitoes. Morphological identification of *An*. *sinensis* is very difficult. Therefore, other *Anophelines* species (*An*. *belenrae*, *An*. *pullus*, and *An*. *lesteri*,) may have been incorrectly identified in this study as *An*. *sinensis*. The use of molecular biological methods could be help improve the accuracy of mosquito identification. The statistical analysis method we used, SARIMA analysis, has the advantage of making a prediction model even if there are not many parameters. However, only linear associations between independent and dependent variables can be used. Therefore, if there is a non-linear relationship between mosquito average abundance and climate variables, the corresponding analysis should be conducted.

It is difficult to accurately forecast that the distribution of adult mosquito abundance. However, predicting future values from past values can improve public health plans. This epidemiological information will be helpful in identifying vector control in place and time and raising local community administrator awareness of potential risk.

Knowledge on the seasonal distribution of mosquitoes provides information about trends and seasonality, allowing the identification of potential high-risk regions for malaria. Time-series analysis of mosquito average abundance and climate factors provides basic information for predicting the occurrence of malaria mosquito.

## Conclusions

In summary, we found that *An*. *sinensis* average abundance was associated with climatic factors in Goyang between 2008 and 2012. The average abundance of mosquito was significantly correlated with the temperature, daily temperature range, duration of sunshine, relative humidity, precipitation, and wind speed at specific time lags. Among these climate variables, grass temperature at time lag0 week and precipitation at time lag1 weeks were fitted the best multivariate model. In this modeling study, climatic variables account for 54.1% of the variance in mosquito average abundance. In addition, our results supported that climatic factors may be important for predicting the occurrence of malaria vectors.

## Supporting information

S1 Dataset (XLSX)

S2 Dataset (XLSX)

S3 Dataset (XLSX)
